# Factors Influencing Infection Anxiety in Korean Male Firefighters Due to COVID-19 Infection Status

**DOI:** 10.3390/healthcare11111623

**Published:** 2023-06-01

**Authors:** Seung-Woo Han, Hyun-Ok Jung

**Affiliations:** 1 Department of Nursing, Kwangju Women’s University, Gwangsan-gu, Gwangju 62396, Republic of Korea; swhan@kwu.ac.kr; 2 The Research Institute of Nursing Science, College of Nursing, Daegu Catholic University, Daegu 42472, Republic of Korea

**Keywords:** COVID-19, infection, job stress, self-management, vaccination, infection anxiety, firefighter

## Abstract

This paper describes descriptive research to identify the effects of job stress, COVID-19 self-care behavior, and COVID-19 vaccination status according to the infection and non-infection of COVID-19 on anxiety about the COVID-19 infection among firefighters in South Korea. Data from 205 firefighters working at 10 fire stations were collected from 26 January to 16 February 2023. The variables used were job stress, COVID-19 self-care behavior, COVID-19 vaccination status, and COVID-19 infection anxiety. The collected data were analyzed using descriptive statistics, *t*-test, one-way ANOVA, Pearson’s correlation coefficient, and multiple linear regression. In subjects who were infected with COVID-19, the factors that significantly affected infection anxiety were job stress (β = 0.247, *p* = 0.011) and self-care behavior (β = 0.343, *p* = 0.011). In subjects who were not infected with COVID-19, the factors that significantly affected infection anxiety were marriage status (unmarried) (β = −0.260, *p* = 0.005) and self-care behavior (β = 0.374, *p* = 0.001). These results demonstrate that the infection anxiety of firefighters should be prevented, and their physical and mental health should be promoted by considering job stress, self-care behavior, and personal environment.

## 1. Introduction

In December 2019, a case of pneumonia of unknown cause occurred in Wuhan, China and was identified as a novel coronavirus infection in January 2020. Due to the rapid spread of the coronavirus worldwide, the World Health Organization declared a pandemic on 11 March 2020 [1]. On 5 May 2023, the World Health Organization lifted the declaration of a public health emergency of international concern (PHEIC) for COVID-19 after 3 years and 4 months. As a result, the international community is accepting it as close to the declaration of the end of the pandemic (endemic). However, uncertainties still exist, such as the emergence of mutant viruses and the occurrence of new mutations due to repeated re-emergence after the initial outbreak of SARS-CoV-2, the virus that causes COVID-19 [2]. Despite the need for an effective treatment strategy, there is still no specific antiviral treatment for COVID-19, and treatment guidelines for COVID-19 vary by country [3]. In South Korea, the first case of COVID-19 occurred on 20 January 2020, and community transmission began on 18 February 2020, including in religious communities and social welfare facilities [4]. In May 2023, there were 68 million confirmed COVID-19 cases and 6.9 million deaths worldwide. South Korea has 31 million confirmed COVID-19 cases, ranking seventh in the world with an incidence rate of 61% per 100,000 people [5], and COVID-19 outbreaks are occurring in various locations in the community.

The COVID-19 pandemic has created unique work, family, and health problems for people around the world [6]. This has caused people all over the world to face physical and psychological stress [7]. A life-threatening pandemic has increased individuals’ anxiety levels, increased avoidance behavior, and disrupted social life [8]. The COVID-19 pandemic, which has caused this social phenomenon, has added more responsibilities for firefighters by preparing protocols based on enhancing public health protection and safety in the already strained fire service system. People’s demand for fire services has increased as they seek to emerge from various crises linked to the COVID-19 pandemic [6]. Fire officials are trying to overcome the COVID-19 pandemic through essential public safety work as front-line workers of COVID-19 [6,9]. Firefighters respond to a variety of hazardous environments, including fire activity and rescue [9,10]. Therefore, firefighters are one of the occupations that are at the highest risk of contracting COVID-19 [11,12]. Firefighters have a high risk of exposure to COVID-19 during patient care and transport, and also have a high risk of transmitting it after being infected [9,13,14]. In addition, police officials working for the safety of the public in the community also have increased physical and psychological fatigue and job stress due to COVID-19 [15].

When exposed to the COVID-19 virus (SARS-CoV-2), firefighters have an increased risk of contracting COVID-19 because of cardiovascular disease due to reduced lung function from frequent fire exposure, rapid cardiac fatigue during acute firefighting, and decreased arterial compliance [9]. 

Firefighters are exposed to many potential sources of occupational stress [10,16]. Their levels of job stress and anxiety increased during the COVID-19 pandemic [17]. Firefighters’ exposure to wildfire smoke during the COVID-19 pandemic has been shown to cause cytotoxicity and inflammation, and these responses increase the risk of COVID-19-related illnesses such as bronchitis and pneumonia. The combined inhalation of the COVID-19 virus (SARS-CoV-2) and wildfire smoke increases the risk of severe COVID-19 symptoms, such as cytokine release syndrome, hypotension, and acute respiratory distress syndrome [18]. 

The COVID-19 RAPID Mental Health Assessment conducted by Drexel University’s Center for Firefighter Injury Research and Safety Trends (FIRST) found that firefighters are under physical and emotional threats. Examples include exhaustion, anxiety, and depression due to fear of spreading the virus to family and friends; changes in eating habits; changes in sleep patterns; workplace quarantine requirements; and lack of access to personal protective equipment [9]. All of these potential occupational stressors of firefighters contribute to an increased susceptibility to COVID-19 infection [9,18]. Increased service demands on fire organizations due to the COVID-19 pandemic may increase job stress in firefighters and increase their risk of developing various mental health problems, along with physical symptoms [10,18,19].

As the number of confirmed COVID-19 cases increases, susceptible groups such as firefighters develop awareness or anxiety about virus transmission [4]. They then change their personal hygiene behavior, e.g., by wearing masks and by social distancing. However, despite these changes, firefighters can still be exposed to the virus through contact with an infected person. South Korean firefighting authorities have recognized this risk of infection, and to reduce the risk to firefighters, have mandated an increased use of personal protective equipment, and have enacted policies and procedures such as COVID-19 vaccination, wearing masks, social distancing, frequent cleaning and disinfection of surfaces, and frequent hand washing. South Korea has also prioritized COVID-19 vaccines as a public health tool to reduce the risk of transmission, and frontline workers such as firefighters have been categorized as priority vaccine recipients [20].

By recognizing the practical demands faced by firefighters during the COVID-19 pandemic, it can be seen how firefighters’ ability to cope with the crisis has a significant effect on their personal physical and psychological health [18]. Therefore, this study aims to identify the effects of job stress, COVID-19 self-management behaviors, and vaccination status on COVID-19 infection anxiety, which can promote susceptibility to behavior change in response to COVID-19 by distinguishing between COVID-19-infected and non-infected firefighters; this study also aims to provide a basis for promoting the physical and mental health of firefighters. 

Furthermore, in the event of an infectious disease such as COVID-19, we would like to suggest an effective plan for care and support programs. Based on the purpose of this study, the hypotheses are as follows:

**Hypothesis 1.**  *Job stress will affect the COVID-19 infection anxiety of both infected and non-infected firefighters.*

**Hypothesis 2.**  *COVID-19 self-care behavior will affect the COVID-19 infection anxiety of infected and non-infected firefighters.*

**Hypothesis 3.**  *COVID-19 vaccination status will affect the COVID-19 infection anxiety of infected and non-infected firefighters*.

## 2. Materials and Methods

### 2.1. Research Design

This is a descriptive research study that uses a self-report survey method to identify the effects of job stress, COVID-19 self-care behaviors, and vaccination status on COVID-19 infection anxiety among firefighters in Korea based on the COVID-19 repair model and infection transmission mode prediction model considering behavioral changes [4].

### 2.2. Study Subjects

The subjects of this study sampled fire stations in six major cities (D, K, M, O, S, and Y cities). Specifically, on-site firefighters working in rescue and emergency services were targeted, excluding office firefighters. The subjects of this study were randomly selected by visiting 10 fire departments in four major cities in South Korea. The number of subjects was calculated using the G*Power 3.1 program to ensure the power of the study, and the minimum convenience sample size required for multiple regression with a significance level (α) of 0.05, power (1 − β) of 0.70, effect size of 0.15, and 9 independent variables was 95 subjects. Considering this required simple size, a total of 220 copies of the questionnaire were distributed, taking into account the dropout rate due to non-return of questionnaires and insincere responses. After excluding 15 copies with many non-responses or insincere responses, the final number of study participants was 205 (103 people who have experienced COVID-19 (infectees) and 102 people who have not experienced COVID-19 (non-infectees). 

The specific selection criteria were as follows: (1) common condition: men who have been working as firefighters for more than one year recently, and who voluntarily agreed to participate in the study after it was explained to them; (2) infectee condition: those who have been notified of COVID-19 infection by a public health center or hospital since the outbreak of COVID-19 in Korea and have experienced quarantine; (3) non-infectee condition: those who have not been notified of COVID-19 infection by a public health center or hospital since the outbreak of COVID-19 in Korea. 

### 2.3. Research Tools

To determine job stress, we used a shortened version of the Korean Job Stress Scale [21]. The scale consists of seven subscales with 24 questions, including 4 on job demand, 4 on insufficient job control, 3 on interpersonal conflict, 2 on job insecurity, 4 on occupational system, 3 on lack of reward, and 4 on occupational climate. Each response uses a 4-point Likert scale from 1 (Very true) to 4 (Not at all), so a high score indicates high job stress. In this study, Cronbach’s α score was 0.85. 

To determine COVID-19 self-care behavior, we adapted a published scale on self-care in COVID-19 [22]. It consists of 20 items in five sub-scales; this includes 5 items of “individual protective measures”, 4 items of “social distancing”, 3 items of “environmental disinfection”, 4 items of “psychological wellbeing”, and 4 items of “healthy lifestyle”. Each response is rated on a 5-point Liker scale from 1 (Never) to 5 (Always), so a high total score indicates good self-care. In this study, Cronbach’s α score was 0.87. 

To determine COVID-19 vaccination status, we asked the subjects to respond to the following two statements: “I have been vaccinated (for COVID-19) to date” and “I have not been vaccinated (for COVID-19) to date”. Question 1 was scored as 1 (Yes) and 0 (No), and question 2 was scored as 1 (No) and 0 (Yes), with higher scores indicating higher vaccination status. In this study, the Cronbach’s α score was 0.80. 

To determine COVID-19 infection anxiety, we adapted and modified a Swine Flu Anxiety Scale [23]. The tool consists of nine items that address perceived likelihood of infection, spread, perceived severity of infection, and avoidance of certain places and people due to infection. Each item is rated on a 5-point Liker scale ranging from 1 (Very little) to 5 (Very much), so a high total score indicates high COVID-19 infection anxiety. In this study, Cronbach’s α score was 0.83. 

### 2.4. Data Collection

All procedures performed in this study were in accordance with the ethical standards of the institutional research committee. Data were collected from 26 January 2023 to 16 February 2023 in accordance with the approved protocol. To ensure that the results of this study were generalizable, we visited a total of 10 fire stations in four large cities in South Korea. We explained the purpose and procedure of the study to the person in charge, obtained permission and cooperation to conduct the study, and issued a recruitment notice to select participants. We personally explained the purpose of the study, ensured anonymity and confidentiality, and asked the participants to complete the questionnaire after receiving their written consent to participate voluntarily. If a participant was infected with COVID-19, we checked the infection notification paper or text from the health center or hospital.

### 2.5. Data Analysis

The collected data were analyzed using the SPSS/WIN 23.0 program. The distributions of general characteristics of the subjects were expressed as real numbers and percentages. The differences of COVID-19 infection anxiety according to the general characteristics of the subjects were analyzed using *t*-tests and one-way ANOVA. The degree of job stress, COVID-19 self-care behavior, vaccination behavior, and infection anxiety were expressed as mean and standard deviation. The correlations between COVID-19 infection anxiety and related variables were quantified using the Pearson correlation coefficient. The assumptions of regression analysis for independent variables were multicollinearity, residual, and singular value, and the influence factors on COVID-19 infection anxiety were quantified using multiple linear regression. 

## 3. Results

### 3.1. Differences in Infection Anxiety between COVID-19 Infectee and Non-Infectee by General Characteristics

The ages of the subjects were “under 40 years old” for 57.3% (59) of the infectees and 59.8% (61) of the non-infectees; the number of years of work was “1-10 years” for 62.1% (64) of the infectees and 65.7% (67) of the non-infectees; the marital status was “married” for 64.1% (66) of the infectees and 52.9% (54) of the non-infectees; the chronic diseases were “do not have” for 93.2% (96) of the infectees and 88.2% (90) of the non-infectees; and subjective health was “very good” for 76.7% (79) of the infectees and 67.7% (69) of the non-infectees. As a result of confirming the difference in the infection anxiety according to the general characteristics of the subjects, there was a statistically significant difference (*t* = −3.07, *p* = 0.003) in the “marriage” group of the non-infectees. In other words, married people who were not infected had higher infection anxiety than unmarried people (Table 1).

### 3.2. Job Stress, Self-Care Behavior, Vaccination Status, and Infection Anxiety by COVID-19 Infection Status

The mean score (±SD) of job stress was 2.10 (±0.31) among the infectees and 2.12 (±0.29) among the non-infectees, and the mean score of self-care behavior was 3.29 (±0.52) among the infectees and 3.26 (±0.54) among the non-infectees. The mean score of vaccination status was 1.00 (±0.05) among the infectees and 0.99 (±0.10) among the non-infectees, and the mean score of infection anxiety was 3.50 (±0.60) among the infectees and 3.45 (±0.62) among the non-infectees. These differences were not statistically significant (Table 2).

### 3.3. Correlations between Study Variables

A significant positive correlation was detected between the self-care behaviors for both the COVID-19 infectees (r = 0.298, *p* = 0.002) and non-infectees (r = 0.387, *p* < 0.001) (Table 3).

### 3.4. Factors Influencing COVID-19 Infection Anxiety

Job stress (β = 0.247, *p* = 0.011) and self-care behaviors (β = 0.343, *p* = 0.011) were significantly affected by infection anxiety among the COVID-19 infectees. Marital status (unmarried) (β = −0.260, *p* = 0.005) and self-care behaviors (β = 0.374, *p* = 0.001) influenced infection anxiety in the non-infectees. Multicollinearity was analyzed to determine the correlations among the domain-specific indices. Multicollinearity is considered to be present when the Tolerance Limit (TL) is > 0.1 and the Variance Inflation Factor (VIF) is > 10. In this study, the TL ranged from 0.937 to 0.975 in the infectees, and from 0.932 to 0.978 in the non-infectees, and the VIF ranged from 1.025 to 1.067 in the infectees, and from 1.023 to 1.072 in the non-infectees; these results indicate that multicollinearity was not a problem. The Durbin–Watson was 2.069 for the infectees and 2.080 for the non-infectees; these results indicate that the residuals were mutually independent, i.e., that the results were not serially autocorrelated (Table 4).

As a result of verifying the hypothesis based on this study, job stress and COVID-19 self-care behavior affected COVID-19 infection anxiety in firefighters infected with COVID-19. Hypotheses 1 and 2 were accepted. However, Hypothesis 3 was rejected because the COVID-19 vaccination status did not affect COVID-19 infection. For uninfected firefighters, only COVID-19 self-care behavior affected COVID-19 infection anxiety. Therefore, Hypothesis 2 was accepted, and Hypotheses 1 and 3 were rejected.

## 4. Discussion

COVID-19 has caused higher anxiety rates than previous infectious diseases. Severe acute respiratory syndrome, influenza, and Ebola caused infection anxiety rates ranging from 3.2% to 12.6% despite high mortality rates, because the infection rates were low or the disease was quickly contained [24]. In contrast, due to high infection rates, high mortality rates, and the emergence of mutant viral strains, the recurrent epidemic of COVID-19 has caused an infection anxiety rate of 31.9%, which is more than three times higher than those of previous respiratory infectious diseases [25]. In this study, we identified the factors that affect infection anxiety among firefighters in South Korea according to the presence of COVID-19 infection. 

It was found that the job stress of firefighters infected with COVID-19 had a significant effect on infection anxiety. These results are similar to a study that found that when COVID-19-infected tourism employees received more direct risk support for protection (e.g., information about COVID-19, infection control) than non-infected employees, the organizational commitment of the infected employees increased, and their job stress decreased as a result of positive behaviors toward organizational support [12]. In addition, during the COVID-19 pandemic in Japan, a study on factors affecting the health-related quality of life of firefighters was presented. As a result of the study, firefighters suspected of having COVID-19 infection had significantly lower physical health scores and higher levels of stress and anxiety than firefighters without a history of suspected infection. High levels of stress and anxiety develop into subjective physical pain symptoms, which are supported by the results that have affected job performance [12]. Recently, Korea has been putting a lot of effort into stress intervention for firefighters through various programs such as the Happy Arts Therapy program. Happy Arts Therapy applies art psychotherapy, music therapy, and group art psychotherapy. Close cooperation between countries will be needed to apply these programs to Western firefighters in the future [26]. 

The results in this study may occur because COVID-19-infected firefighters experienced direct exposure to COVID-19 infection in their roles of rescue, paramedicine, and firefighting, so they recognized the need to adopt a systematic risk management system for infectious diseases (e.g., social and physical distancing when in contact with community members, wearing masks, cleaning and disinfecting firefighting equipment, daily screening for COVID-19 symptoms and temperature) to reduce job stress [27]. Overseas studies show that about 12.8% of healthcare workers in Serbia have severe COVID-19 anxiety prevalence [28]. In addition, in a study conducted on teachers, an occupation vulnerable to infection in Spain, about 15.7% showed a severe COVID-19 prevalence [29]. As shown in previous studies in various countries, it can be seen that serious infection anxiety is observed in various occupations. Therefore, it is necessary to lower the level of job stress by strengthening the care and support system (e.g., developing an infectious disease response plan, training for infectious diseases, ensuring flexible working conditions, putting in alternative workers in case of infection of essential workers, increasing access to paid sick leave, and risk benefits for people exposed during the pandemic) for COVID-19-infected fire officials.

Marital status (never married) significantly affected COVID-19 infection anxiety among firefighters who had not contracted COVID-19. These results show that in a study of medical workers caring for patients at the forefront of the COVID-19 pandemic, there were more unmarried non-COVID-19 patients than married ones [30]. In the case of unmarried people, the quality of life in the physical, social, psychological, and environmental domains related to COVID-19 was lower than that of married people. These findings are not surprising given that the study population is entirely male, and they spend time in two different environments, i.e., either working with male coworkers while at the fire station or spending time with their wives at home for married firefighters. These two environments can buffer against job stress [31]. Marital status may have influenced infection anxiety among firefighters who are unmarried and had not been infected, because they spend more time at the fire station with coworkers who are exposed to COVID-19 than at home [30]. By observing firefighters who live in an accommodation close to the fire station and often share a joint meal with the common sleeping room [11], it is thought that marital status (unmarried) has affected infection anxiety.

According to a survey of 53,557 Korean firefighters on the work status of Korean firefighters in the COVID-19 pandemic, 24,507 firefighters had experience in responding to COVID-19, accounting for 45.76% of the survey group [32]. Self-care behaviors affected COVID-19 infection anxiety among both the infectees and non-infectees. SARS-CoV-2 is transmitted directly from an infected individual through the inhalation of respiratory droplets, and is indirectly transferred from contaminated hands or surfaces to the mouth, nose, or eyes [33]. The World Health Organization developed guidelines to improve the knowledge, attitudes, and behaviors about COVID-19, and emphasized that these guidelines can slow the spread of COVID-19 infection [34]. These guidelines include individual protective measures (e.g., handwashing, avoiding touching your eyes, nose, and mouth with your hands, wearing a mask, and using disposable gloves in risky areas), environmental disinfection (e.g., using disinfectants, disinfecting shared objects and surfaces, and ventilating rooms), social distancing (e.g., avoiding shaking hands and hugging, staying at least 1 m away from people, staying home, etc.), and maintaining wellbeing (e.g., relieving stress, engaging in regular physical activity, maintaining a healthy lifestyle such as not drinking or smoking, and maintaining a balanced nutrition) [22]. Adherence to self-care behaviors can help to reduce COVID-19 transmission by clearing coronavirus and preventing droplet transmission [35], and is therefore thought to be a factor in infection anxiety in both infectees and non-infectees. In addition, it is believed that the higher the COVID-19 self-management behavior, the lower the fear of spreading the virus to family and friends which threatens the physical and mental health of fire officials. Furthermore, it is thought that self-management behavior emerged as an infection anxiety factor for COVID-19 due to the increased probability [6] of the self-management of eating habits and sleep patterns and complying with quarantine requirements at work.

The results of this study show that there was no statistically significant difference in COVID-19 infection anxiety for both COVID-19-infected and non-infected firefighters according to the COVID-19 mathematical modeling and infection transmission pattern prediction model considering behavioral changes [4]. This study detected no statistically significant difference in COVID-19 infection anxiety between infectees and non-infectees as a susceptible group for infection transmission. These results indicate that as the number of confirmed COVID-19 cases increased, firefighters with anxiety about virus infection changed their behavior by adopting self-management behaviors such as hand washing, mask wearing, and social distancing to try to block the transmission of infection [30]. Results also indicate that job stress for infectees and marital status (unmarried) for non-infectees further promoted COVID-19 infection anxiety. 

The theoretical meaning of this study was based on the “prediction of COVID-19 transmission dynamics using mathematical model considering behavioral changes” for both infected and non-infected firefighters. This prediction was that job stress, COVID-19 self-care behavior, and COVID-19 vaccination behavior increased COVID-19 infection anxiety in the process of moving to behavioral sensitivity. However, the practical meaning of this study is that in the process of moving firefighters to behavioral change susceptibility, job stress for firefighters infected with COVID-19 and marital status (single) for firefighters without COVID-19 infection further promote anxiety about COVID-19 infection. Therefore, it is necessary to consider job stress, personal environment such as marital status, and self-care behavior variables when providing care and support programs related to infectious diseases to firefighters in the future. 

This study has limitations. First, it considered only male firefighters; future studies should include female firefighters and various demographic variables to conduct a substantial and multifaceted analysis of infection anxiety. Second, domestic and international research has not evaluated infection anxiety among firefighters within the last three years. Therefore, future research should focus on infection anxiety in high-risk occupations, including firefighters. This research should be accompanied by the development of various programs to promote infection prevention and counseling on infection anxiety at the national level. Finally, this study has not quantified the characteristics of infection anxiety among firefighters. Future research should quantitatively evaluate the phenomenon of infection anxiety of firefighters in the actual field and incorporate their actual infection anxiety complaints in the research.

Nevertheless, this study is significant in that it was conducted to compare the effect of the presence of infection in a difficult environment that requires personal privacy of COVID-19 infection. We believe that this study can provide basic data that can guide the development of various care and support programs in the future by identifying factors that affect infection anxiety in firefighters with and without COVID-19 infection experience. In addition, the significance of this study is that the subjects of this study were firefighters working at 10 fire stations in Korea, and the environmental factors for the spread of COVID-19 were considered by collecting subjects who were not limited to one region.

## 5. Conclusions

Fire officials are always performing the most physically and mentally difficult tasks in a high-risk environment. Firefighters perform essential public safety work and have continued that work despite the challenges of COVID-19. Firefighters had higher levels of job stress and infection anxiety during the COVID-19 pandemic than before due to their frequent contact with the community, including transporting COVID-19 patients [16]. Job stress and self-care behaviors among infectees, and marital status (unmarried) and self-care behaviors among non-infectees had significant effects on infection anxiety. However, COVID-19 vaccination status in the infected group did not appear to affect COVID-19 infection anxiety. In the uninfected group, job stress and COVID-19 vaccination status did not appear to have an effect on COVID-19 infection anxiety. In particular, COVID-19 vaccination status was not affected in both the infected and non-infected groups, so it will be necessary to find out the cause of this in more diverse ways in the future. Additionally, when providing COVID-19-related care and support programs to firefighters, it is necessary to promote physical and mental health in consideration of job stress, self-management behavior, and personal environment (e.g., marital status).

## Figures and Tables

**Table 1:** Distribution by general characteristics of subjects.

Characteristics	Category	Infected (n = 103)				Non-Infected (n = 102)			
		n (%)	M (SD)	t/F	*p*	n (%)	M (SD)	t/F	*p*
Age	<40	59 (57.3)	3.45 (0.64)	−0.96	0.340	61 (59.8)	3.38 (0.69)	−1.47	0.145
	≥40	44 (42.7)	3.57 (0.56)			41 (40.2)	3.56 (0.50)		
Working period	1–10 years	64 (62.1)	3.47 (0.65)	1.08	0.358	67 (65.7)	3.37 (0.67)	1.08	0.358
	11–20 years	28 (27.2)	3.54 (0.50)			19 (18.6)	3.63 (0.49)		
	21–30 years	10 (9.7)	3.56 (0.63)			13 (12.8)	3.54 (0.53)		
	More than 31 years	1 (1.0)	3.78 (-)			3 (2.9)	3.67 (0.68)		
Marriage	Unmarried	37 (35.9)	3.38 (0.71)	−1.45	0.152	48 (47.1)	3.26 (0.64)	−3.07	0.003
	Married	66 (64.1)	3.57 (0.53)			54 (52.9)	3.62 (0.56)		
Chronic disease	Have	7 (6.8)	3.57 (0.39)	0.32	0.746	12 (11.8)	3.44 (0.58)	−0.10	0.921
	Not have	96 (93.2)	3.49 (0.62)			90 (88.2)	3.45 (0.63)		
Subjective healthstatus	Healthy, very good	79 (76.7)	3.54 (0.60)	0.35	0.703	69 (67.7)	3.45 (0.60)	0.35	0.703
	Normal	23 (22.3)	3.34 (0.63)			31 (30.4)	3.47 (0.68)		
	Unhealthy, not good	1 (1.0)	3.78 (-)			2 (2.0)	3.39 (0.86)		

**Table 2:** Degree of COVID-19 infection anxiety, job stress, COVID-19 self-care behavior, and COVID-19 vaccination status.

Variable	Infected (n = 103)	Non-Infected (n = 102)	t	*p*
	M (SD)	M (SD)		
COVID-19 infection anxiety	3.50 (0.60)	3.45 (0.62)	0.55	0.581
Job stress	2.10 (0.31)	2.12 (0.29)	−0.29	0.772
COVID-19 self-care behavior	3.29 (0.52)	3.26 (0.54)	0.41	0.684
COVID-19 vaccination status	1.00 (0.05)	0.99 (0.10)	0.45	0.652

**Table 3:** Correlation between study variables.

Variable	COVID-19 Infection Anxiety	
	Infected (n = 103)	Non-Infected (n = 102)
	r (*p*)	r (*p*)
Job stress	0.170 (0.086)	−0.045 (0.650)
COVID-19 self-care behavior	0.298 (0.002)	0.387 (<0.001)
COVID-19 vaccination status	0.082 (0.409)	0.073 (0.469)
COVID-19 infection anxiety	1	1

**Table 4:** Factors affecting COVID-19 infection anxiety.

**Variable**	**B**	**SE**	**β**	**t**	***p***
Infected (n = 103)					
(Constant)	0.580	1.261	-	0.46	0.646
Job stress	0.485	0.187	0.247	2.59	0.011
COVID-19 self-care behavior	0.396	0.111	0.343	3.58	0.001
COVID-19 vaccination status	0.599	1.153	0.049	0.52	0.605
Durbin–Watson = 2.069, Tolerance Limit = 0.937~0.975, VIF = 1.025~1.067, R^2^ = 0.2482, Adjusted R^2^ = 0.2223, F = 5.74, *p* = 0.001					
**Variable**	**B**	**SE**	**β**	**t**	***p***
Non-infected (n = 102)					
(Constant)	1.836	0.873	-	2.10	0.038
Marriage					
Unmarried	−0.324	0.112	−0.260	−2.88	0.005
Married (ref)					
Job stress	0.066	0.202	0.030	0.33	0.744
COVID-19 self-care behavior	0.428	0.106	0.374	4.06	0.001
COVID-19 vaccination status	0.233	0.570	0.037	0.41	0.684
Durbin–Watson = 2.080, Tolerance Limit = 0.932~0.978, VIF = 1.023~1.072, R^2^ = 0.3227, Adjusted R^2^ = 0.2906, F = 6.95, *p* < 0.001					

## Data Availability

Not applicable.
